# Editorial for Special Issue “Multidrug-Resistant Pathogens”

**DOI:** 10.3390/microorganisms8091383

**Published:** 2020-09-10

**Authors:** Despoina Koulenti, Paraskevi C. Fragkou, Sotirios Tsiodras

**Affiliations:** 1 UQ Centre for Clinical Research, Faculty of Medicine, The University of Queensland, Brisbane, QLD 4072, Australia; 2 2nd Critical Care Department, Attikon University Hospital, 12462 Athens, Greece; 3 4th Department of Internal Medicine, Attikon University Hospital, 12462 Athens, Greece; evita.fragou@gmail.com (P.C.F.); sotirios.tsiodras@gmail.com (S.T.)

The era of injudicious use of antibiotics in both humans and animals has led to the selection of multidrug-resistant (MDR) pathogens, which in turn has left the medical community with limited therapeutic options. MDR infections are associated not only with significant morbidity and mortality, but also they represent a huge economic burden for the healthcare system globally [1]. Importantly, in addition to MDR infections associated with the healthcare setting, which represent the major concerns at present, MDR organisms are increasingly identified in community-acquired infections as well as in animals. This phenomenon signifies that excessive use of antibiotics has led to the universal spread of resistant pathogens, affecting humans both directly and indirectly though the food chain. Hence, further research is urgently needed to guide the best approaches and practices for the prevention and control of MDR infections, as well as to investigate the role of novel and old antimicrobials in the fight against MDR pathogens. Moreover, the development and validation of rapid and reliable diagnostic techniques will enable the fast identification of resistance patterns that would facilitate the prompt implementation of targeted treatment and infection control measures. The 22 articles in the Special Issue on “Multidrug-Resistant Pathogens” present an in-depth and multifaceted approach to different aspects of infections caused by MDR strains, including epidemiological aspects, prevention strategies, as well as the role of novel and old “re-discovered” antimicrobials and other emerging therapeutic options.

MDR pathogens are usually implicated in nosocomial infections. Rouzé et al. conducted an ancillary analysis of a prospective multicenter study on the impact of chronic obstructive pulmonary disease (COPD) in ventilator-associated lower respiratory tract infections, and found that the rate of MDR bacteria was not significantly different between COPD and non-COPD patients, while *Escherichia coli* and *Stenotrophomonas maltophilia* were significantly more frequent in COPD patients [2]. A retrospective five-year single cohort observational study on the epidemiology of MDR infection in 73 oncological patients by Perdikouri et al. showed that the most frequently isolated pathogens were carbapenem-resistant *Klebsiella pneumoniae*, methicillin-resistant *Staphylococcus aureus* (MRSA) and carbapenem-resistant *Acinetobacter baumannii*, while infection-associated mortality was as high as 32% [3]. Caneiras et al. conducted a multicenter retrospective study of 81 *K. pneumoniae* strains isolated from patients with community-acquired or hospital-acquired urinary tract infections; the authors concluded that all 31 hospital-acquired isolates were extended-spectrum β-lactamase (ESBL) producers and had a multidrug resistant profile as compared to the community strains, despite that *bla* genes were also detected in 12% of the community strains [4]. Although MDR pathogens are primarily associated with nosocomial infections, evidence shows that bacteria such as MDR *Pseudomonas aeruginosa*, ESBL *Enterobacteriaceae* and MRSA are increasingly isolated by patients with severe community-acquired pneumonia, as demonstrated by Cillóniz et al. [5].

Even though the use of colistin has been abandoned for years, due to the small therapeutic range and high potential for toxicity, its utility as a last-resort agent for MDR infections has been revisited. Colistin dosing schemes, especially in critically ill patients, remain ambiguous. However, the study by Ehrentraut et al. shed light on the pharmacokinetics of colistin in this patient group. The authors evaluated the efficiency of the current guidelines’ recommendations by using high resolution therapeutic drug monitoring of colistin. The authors analyzed plasma levels of colistin and its pro-drug colisthimethate sodium (CMS) in 779 samples, drawn from eight intensive care unit (ICU) patients with pan-drug resistant (PDR) infections [6]. This study found that CMS levels did not correlate with colistin levels and over- or under-dosing occurred regardless of renal function and the mode of renal replacement therapy, while colistin elimination half-time appeared to be longer than previously reported [6].

The resurgence of colistin in the antimicrobial armamentarium against MDR infections has led to the emergence of resistance among these pathogens. Papathanakos et al. conducted a retrospective observational study in ICU patients with colistin-resistant extensively drug-resistant (XDR) *A. baumannii* bacteremia to assess the mortality and to compare the characteristics of bloodstream infections by colistin-resistant and colistin-sensitive *A. baumannii* strains [7]. The authors found a mortality rate of 100% in colistin-resistant *A. baumannii* bacteremia, which was significantly higher than in patients with susceptible strains; additionally, the majority (69%) of colistin-resistant *A. baumannii* bacteremias led to fulminant septic shock and death within three days of symptoms’ onset [7]. 

Regarding the epidemiology of colistin-resistant MDR strains, Elbediwi et al., in their systematic review and meta-analysis, found an overall average prevalence of 4.7% (0.1–9.3%) among 47 countries and across six continents, with China reporting the highest numbers of mobilized colistin resistance (*mcr*)-positive strains in bacteria isolated from humans, animals, the environment and food products [8]. *E. coli* (54%) isolated from animals (52%) and harboring an IncI2 plasmid (34%) were the bacteria with the highest prevalence of *mcr* genes, while the estimated prevalence of *mcr*-1 pathogenic *E. coli* was higher in food-animals than in humans and food products, suggesting a possible contribution of foodborne transmission of such strains [8]. Additionally, Wyrsch et al. identified two atypical Z/I1 hybrid plasmids (pSTM32-108 and pSTM37-118) hosting antimicrobial resistance and virulence-associated genes within endemic pathogen *Salmonella enterica* serovar Typhimurium 1,4,[5],12:i:-, isolated in Australian swine production facilities, and demonstrated that these plasmids are relatives of close relatives of two plasmids isolated from *E. coli* of human and bovine origin many years ago [9]. The epidemiology of plasmid-mediated colistin resistance in *Salmonella enterica* serovars was reviewed by Lima et al. who concluded that *mcr*-like genes are carried in conjugative plasmids that spread among bacterial populations and plasmid-mediated colistin resistance genes may reach human microbiota through the food chain [10]. Interestingly, Harada et al. investigated the resistance profile of *Serratia* spp. and *Citrobacter* spp. in companion animals, and found that 34.8% of *Citrobacter* isolates were extended-spectrum cephalosporin-resistant, while no resistant profiles were detected in *Serratia* strains [11].

Although horizontal gene transfer plays a key role in the dissemination of antimicrobial resistance acquisition, other mechanisms such as competence and natural transformation may also contribute to resistance development in some strains, such as *A. baumannii*. Domingues et al. in their study with 22 *Acinetobacter* isolates demonstrated that natural competence is common among clinical isolates of *Acinetobacter* spp. and therefore it is likely an important contributor of resistance acquisition in this genus [12]. Andrzejczuk et al. studied the prevalence of β-lactam resistance and *bla* genes among 87 *Haemophilus parainfluenzae* isolates from respiratory microbiota of adult patients, and demonstrated that among the 57 (65.5%) beta-lactam-resistant isolates, 63.2% encoded *bla* genes with *bla_TEM-1_* being the most frequent identified gene [13]. 

In addition to colistin, other treatment options, including older antibiotics as well as novel agents and approaches, have been studied. Fragkou et al. reviewed the role of minocycline in the treatment of nosocomial infections caused by MDR, XDR and PDR *A. baumannii*, concluding that it represents a plausible treatment option for these strains but further studies are needed [14]. Moreover, Feehan and Garcia-Diaz reviewed the evidence on novel non-antibiotic-based microbiome-modifying bacterial therapies, such as fecal microbiota transplantation (FMT), prebiotics and probiotics in the management of infections and gut colonization caused by MDR organisms, and highlighted the need for further development of other therapies, such as bacteriophages, lytic enzymes, novel cleaning techniques, repurposed drugs with antibiotic activity, and bacterial byproducts [15]. Naskar and Kim reviewed the role of different types of nanomaterials, such as metallic and organic nanoparticles, both as antimicrobial carriers and as potential alternative therapeutic options for MDR pathogens, but clinical research data on nanomaterial-based antibacterial approaches are still limited [16]. Koulenti et al. reviewed the evidence on mechanism of action, pharmacokinetics, microbiological spectrum, efficacy and safety profiles of novel branded antibiotics against MDR Gram-positive pathogens, namely ceftobiprole, ceftaroline, telavancin, oritavancin, dalbavancin, tedizolid, besifloxacin, delafloxacin, ozenoxacin, omadacycline and lefamulin [17,18]. Moreover, the same authors conducted another thorough review of all emerging agents that are currently under clinical development in phase I, II and III clinical trials for the treatment of MDR Gram-positive organisms including novel β-lactams, oxazolidinones, quinolones, amininoglycosides, ketolides, defensin mimetic drugs, a new bacterial topoisomerase II inhibitor, FabI inhibitors under development, new polymyxin derivatives as well as bacteriophages and monoclonal antibodies [19]. Finally, Russo et al. conducted an experimental study using predatory bacteria (*Bdellovibrio bacteriovorus*) against *Yersinia pestis* inoculated in the lungs of mice, and demonstrated that three doses of *B. bacteriovorus* reduced the number of colony-forming units by 86% within 24 h of infection, thus posing another possible therapeutic strategy for severe Gram-negative infections [20].

The prevention of the spread of MDR organisms remains one of the most important strategies in the management of these pathogens. Cotoia et al. reviewed the evidence from 27 original articles regarding preventative strategies for MDR-induced ventilator-associated pneumonia; the authors reported numerous preventative measures with the most convincing evidence coming from those that prevent oropharyngeal tract colonization with MDR strains and their descent into the respiratory tract [21]. Finally, one of the most important determinants of resistance development is the impact of antibiotics on gut microbiota; thus, Pilmis et al. reviewed various strategies to limit these effects on intestinal microbiome or to cure dysbiosis such as antimicrobial stewardship, action on residual antibiotics at colonic level, prebiotics, probiotics and FMT, concluding that more data are needed before we can draw robust outcomes [22].
